# Increased prediction value of biomarker combinations for the conversion of mild cognitive impairment to Alzheimer’s dementia

**DOI:** 10.1186/s40035-020-00210-5

**Published:** 2020-08-03

**Authors:** Aonan Zhao, Yuanyuan Li, Yi Yan, Yinghui Qiu, Binyin Li, Wei Xu, Ying Wang, Jun Liu, Yulei Deng

**Affiliations:** 1 Department of Neurology and Institute of Neurology, Ruijin Hospital affiliated to Shanghai Jiao Tong University School of Medicine, Shanghai, China; 2 Department of Neurology, RuiJin Hospital/LuWan Branch, School of Medicine, Shanghai Jiaotong University, Shanghai, China

**Keywords:** Alzheimer’s disease, Mild cognitive impairment, *Olfactory function*, Neuronal-derived exosomes

## Abstract

**Background:**

Progression of mild cognitive impairment (MCI) to Alzheimer’s disease (AD) dementia can be predicted by clinical features and a combination of biomarkers may increase the predictive power. In the present study, we investigated whether the combination of olfactory function and plasma neuronal-derived exosome (NDE) Aβ_1–42_ can best predict progression to AD dementia.

**Methods:**

87 MCI patients were enrolled and received the cognitive assessment at 2-year and 3-year follow-up to reevaluate cognition. In the meanwhile, 80 healthy controls and 88 AD dementia patients were enrolled at baseline as well to evaluate the diagnose value in cross-section. Olfactory function was evaluated with the sniffin sticks (SS-16) and Aβ_1–42_ levels in NDEs were determined by ELISA. Logistic regression was performed to evaluate the risk factors for cognitive decline in MCI at 2-year and 3-year revisits.

**Results:**

In the cross cohort, lower SS-16 scores and higher Aβ_1–42_ levels in NDEs were found in MCI and AD dementia compared to healthy controls. For the longitudinal set, 8 MCI individuals developed AD dementia within 2 years, and 16 MCI individuals developed AD dementia within 3 years. The two parameter-combination of SS-16 scores and Aβ_1–42_ level in NDEs showed better prediction in the conversion of MCI to AD dementia at 2-year and 3-year revisit. Moreover, after a 3-year follow-up, SS-16 scores also significantly predicted the conversion to AD dementia, where lower scores were associated with a 10-fold increased risk of developing AD dementia (*p* = 0.006). Similarly, higher Aβ_1–42_ levels in NDEs in patients with MCI increased the risk of developing AD dementia by 8.5-fold (*p* = 0.002).

**Conclusion:**

A combination of two biomarkers of NDEs (Aβ_1–42_) and SS-16 predicted the conversion of MCI to AD dementia more accurately in combination. These findings have critical implications for understanding the pathophysiology of AD dementia and for developing preventative treatments for cognitive decline.

## Introduction

Mild cognitive impairment (MCI), the stage between normal aging and Alzheimer’s disease (AD) dementia, is associated with a higher risk of dementia [1, 2]. A recent meta-analysis indicated that about 45% of MCI patients maintained stable, whereas 28% progressed to AD and 15% return to normal status without recurrence [3]. Recent work has aimed to improve the detection of the early stages in AD dementia and improve the methods used to identify individuals with MCI who are at high risk of developing AD dementia.

Olfactory dysfunction has been identified in patients with AD dementia [4]. In a longitudinal study, olfactory impairment was used as a biomarker for diagnosing MCI and AD dementia, predicting the progression of AD dementia in normally-aging individuals [5]. However, odor identification tests are not specific to AD dementia and may also be impaired in Parkinson’s disease and other forms of dementia [6]. Given that olfactory function appears to be altered across neurodegenerative diseases and is also affected by smoking habits and respiratory diseases [7], more sensitive and specific biomarkers are needed to assist odor identification tests as a clinical diagnostic role of AD dementia.

Amyloid beta (Aβ), AD-specific pathological changes, are deposited in the olfactory bulb or other brain regions related to olfactory function [8]. *Aβ*_*1–40*_ and *Aβ*_*1–42*_ in cerebrospinal fluid (CSF) have consistently been shown to predict the conversion from MCI to AD dementia [9]. However, the measurement of CSF biomarkers is invasive and discrepant findings have been reported when attempting to use a combination of plasma proteins to predict AD dementia progression in stable MCI patients [10]. Hence, a more accurate measurement for *Aβ*_*1–42*_*with* better clinical practicality is in need. Neuronal-derived exosomes (NDEs) in plasma are released from neurons, reported to contain amyloid-beta precursor protein and Aβ, released from the central nervous system (CNS) [11]. Aβ, contained within NDEs, isolated from plasma, accurately predicted the development of AD dementia up to 5 years before AD onset [12] . Therefore, the need to recognize AD dementia at an early or more treatable stage promotes the study of exosome biomarkers. The evaluation of olfactory function combined with Aβ in NDEs may lead to new approaches in predicting the risk of MCI to AD dementia.

However, no studies to date have investigated whether the combination of Aβ and olfactory test can improve predicting the transition risk of MCI to AD dementia. Firstly, we aimed to measure Aβ levels from plasma NDEs and odor identification in AD dementia and MCI groups. Secondly, this longitudinal study focused on patients with MCI who progressed to a probable AD dementia within 3 years after baseline (called MCI converters (MCI-c)) and compared them with clinically stable patients who did not develop to AD dementia (called MCI non-converters (MCI-nc)). The *olfactory test and the neurogenic exosomes (Aβ*_*1–42*_*&Aβ*_*1–40*_*) were performed to investigate baseline differences between MCI converters and non-converters. The purpose of the research was* to establish a predictive model identifying individuals with MCI who are at risk of developing AD dementia. We hypothesized that a combination of the sniffin sticks (SS-16) and plasma NDEs Aβhelped screen and MCI patients at higher risk of cognitive decline, which can benefit from early intervention to prevent the risk of disease progression.

## Subjects and methods

### Study population and clinical profiling

Participants were recruited from the neurology clinic at Ruijin Hospital from October 2015 to May 2019. All volunteers gave their informed, written consent prior to study participation. This study was approved by the Research Ethics Committee of Ruijin Hospital. All patients with AD dementia were diagnosed as probable AD dementia following the National Institute on Aging and Alzheimer’s Association (NIA-AA) diagnostic guidelines for probable AD dementia with support of structural MRI images [13]. To ensure volunteers understood the task, only patients with mild to moderate AD dementia (24 ≥ Mini Mental State Examination (MMSE) ≥ 10) participated on the odor identification tests. MCI with deficits in memory function were diagnosed according to the Mayo Clinic criteria [14, 15]. The criteria include subjective memory complaint corroborated by an informant together with preserved everyday activities, a memory impairment based on a standard neuropsychological test, preserved global cognitive functions and finally the exclusion of dementia. The healthy control subjects were age-, sex-, and education-matched and were recruited from the local community in Shanghai. Inclusion criteria for normal controls required a MMSE score ≥ 28 without any memory-related complaint. Subjects with the presence of dementia or other neurological diseases such as Parkinson’s disease were excluded. Besides, participants were excluded if they have any of the following medical problems: acute diabetic complications, history of acute cerebrovascular accident, history of acute cardiovascular accident, systemic disorders such as malignancy and lupus which were not cured, severe infection, drug abuse or dependency condition and severe psychiatric disorders which were not cured. In this study, we excluded participants with possible factors impairing olfactory function, such as chronic rhinitis, recent upper respiratory infections, and nose surgery.

All participants completed the neuropsychological battery including the MMSE [16], the Montreal Cognitive Assessment (MoCA) [14], Auditory Verbal Learning Test (AVLT) [17], Alzheimer’s Disease Assessment Scale cognitive subscale (ADAS-cog),Zung Self-rating the Anxiety Scale (SAS) and the Zung Self-rating Depression Scale (SDS) [18]. All tests were administered by memory-related specialists with professional training. Experienced neurologists performed all diagnoses based on a thorough review of the patient’s medical history, neurological examinations, laboratory tests and structural MRI results. All participants in the study (*n* = 255) including 80 healthy controls, 87 individuals with MCI and 88 patients with AD dementia completed the SS-16 test and the tests measuring neuronal-derived exosomes at baseline. Follow-up visiting started from January 2017 to May 2019, where the mean follow-up time was 34.7 months. During the 2-year and 3-year follow-ups, patients with MCI were reclassified as MCI converters (MCI-c) or MCI non-converters (MCI-nc) based on whether they had been diagnosed with AD dementia.

### SS-16 assessment

In the study, the SS-16 was selected as an evaluation instrument to verify the sensitivity and feasibility of the odor identification test. The Chinese version of the SS-16 was validated with Chinese patients with Parkinson’s disease in 2012 [19]. Trained specialists, who were blinded to the diagnosis of the volunteer, administered the 16-item odor identification tests. Participants were required to place the odor sticks 2 cm away from their nose and were instructed to smell the stick for 3 s. Following this, volunteers were instructed to identify the odor using a multiple-choice question with 4 possible answers. The time interval between the presentation of each odor was approximately 30 s. Each correct answer was assigned one point and the total score varied from 0 to the highest possible of 16.

### Isolation of neuronal-derived exosomes (NDEs) from plasma

L1 cell adhesion molecule (L1CAM) is a member of cell adhesion molecules primarily expressed in the nervous system and is proved to be a marker on the surface of exosomes that are specifically derived from the neurons [20, 21]. Exosomes were collected from plasma and the content of those originating from neurons (NDEs) were enriched by absorption with the anti-L1CAM antibody. Overall, 500 μL plasma was incubated with thromboplastin-D (Fisher Scientific, Inc., Hanover Park, IL) followed by calcium- and magnesium-free Dulbecco balanced salt solution with protease inhibitor cocktail (Roche Applied Sciences, Inc., Indianapolis, IN) and phosphatase inhibitor cocktail (Pierce Halt, Thermo Scientific, Inc., Rockford, IL). After centrifugation, supernatants were incubated with ExoQuick exosome precipitation solution (EXOQ; System Biosciences, Inc., Mountain View, CA) and resultant suspensions centrifuged at 1500×g for 30 min at 4 °C [22]. Each pellet was re-suspended in 200 μL of distilled water with inhibitor cocktails followed by immunochemical enrichment of exosomes from neural sources.

Total exosome suspensions were incubated with 2 μg of mouse anti-human L1CAM (neural adhesion protein) biotinylated antibody (Abcam, Cambridge, MA, USA) in 50 μL of 3% BSA for 60 min at 20 °C followed by addition of 10 μL of Streptavidin-Plus UltraLink resin (Pierce-Thermo Scientific, Inc.) in 40 μL of 3% BSA and further incubation for 60 min [19]. After centrifugation at 400×g for 5 min at 4 °C, pellets were re-suspended in 50 μL of 0.05-M glycine-HCl (pH 3.0), incubated at 4 °C for 10 min, and re-centrifuged. Each supernatant was transferred to a new Eppendorf tube containing 5 μL of 1-M Tris-HCl (pH 8.0) mixed with 0.50 mL M-PER mammalian protein extraction reagent (Thermo Scientific, Inc.), containing protease and phosphatase inhibitors, and mixed and stored at − 80 °C.

### Quantification of NDE and ELISA assay

L1CAM-positive NDE cargo proteins were quantified by using the human-specific ELISAs for Aβ_1–42_ (Anogen, Ontario, CA), Aβ_1–40_ (Anogen, Ontario, CA) and ExoELISA CD63 Kit (System Biosciences, Inc., Mountain View, CA) in duplicate with verification of bicinchoninic acid (BCA) reagent-based protein quantitation (Thermo Scientific, Inc.) to normalize the relative values for each sample.

L1CAM-positive plasma NDEs were characterized based on size and shape using transmission electron microscopy (TEM). The degree of purity was verified by western blot with positive exosomal marker CD63 (Abcam, Cambridge, MA, USA) and Tsg101 (Abcam, Cambridge, MA, USA) and negative exosomal marker GM130 (Abcam, Cambridge, MA, USA). The size of the samples was directly determined by NTA using a NanoSight LM10 microscope (NanoSight Ltd., Salisbury, UK).

### ApoE ε4 genotype

Genomic DNA was extracted from peripheral blood through the standardized phenol/chloroform extraction method. ApoE ε2/3/4 alleles were determined by the following primers to detect rs7412 and rs429358. Forward primer: AGGAACAACTGACCCCGGTG; Reverse Primer: GCTGCCCATCTCCTCCATCC. All subjects were classified as ApoE ε4 carriers with APOE ε2/ε4, ε3/ε4 and ε4/ε4 or as ApoE ε4 non-carriers with APOE ε2/ε2, ε2/ε3 and ε3/ε3.

### Statistical analysis

Statistical analyses were conducted with SPSS (*version* 19.1; IBM Corp., Armonk, NY). The significance level was set at *p* < 0.05. One-way ANOVAs with the least significant difference (LSD) and post-hoc tests were used to compare differences between the three groups (AD dementia, MCI, and healthy controls). We used chi-squared and split chi-squared tests to identify differences in sex, education levels, smoking status and the accuracy of detecting the 16 odors between the three groups. The Pearson correlation was used to determine the association between Aβ1–42, SS-16 and MMSE/MoCA scores. Receiver operating characteristic (ROC) curves were plotted for SS-16 and Aβ_1–42_ in NDEs by calculating the sensitivity and specificity of their diagnostic power in HC, MCI, AD dementia, MCI-c and MCI-nc [23]. Logistic regression was used to evaluate whether biological variables (SS-16, Aβ_1–42_ and ApoE ε4 status) predicted the conversion to AD dementia in individuals with MCI at 2-year and 3-year follow-up. To assess how SS-16 and Aβ_1–42_ increase the risk of AD dementia conversion, we built a logistic regression model of the ten markers controlling for age, sex and education.

## Results

### Demographic and neuropsychological characteristics

Demographic features and clinical data of healthy controls and patients with AD dementia and MCI are shown in Table 1. There were no significant differences between the three groups based on age, sex, or education levels. Furthermore, we classified the enrolled subjects to be ApoEε4 carriers or ApoE ε4 non-carriers. 39% of AD dementia patients, 17% of MCI patients and 10% of healthy individuals were positive for ApoE ε4. A greater number of patients with AD dementia were ApoEε4 carriers relative to controls (*p* < 0.001, Table 1). 59% of AD dementia patients were maintained on cholinesterase inhibitors with a mean dose of 5.2 mg per day. Compared with MCI and controls, patients with AD dementia had lower scores of MMSE, MoCA, AVLT-SR and AVLT-LR (*p* < 0.001, Table 1), but exhibited a higher ADAS-cog score (*p* < 0.001, Table 1). However, MCI subjects also had lower MMSE, MoCA and AVLT scores relative to controls and greater ADAS-cog scores compared to controls (*p* < 0.05; Table 1). Applied a cutoff score of 1 SD under population mean standardized for age and gender according to AVLT tests [24], 74 out of the enrolled MCI patients were amnestic MCI (aMCI). Olfactory function was further assessed, and scores of SS-16 were significantly lower in AD dementia than in the control and MCI groups (11.2 ± 1.9 for controls; 9.1 ± 2.7 for MCI; 5.9 ± 2.7 for AD dementia; *p* < 0.001; Table 1). Moreover, lower SS-16 scores were observed in MCI subjects than in healthy controls (*p* < 0.001).

**Table 1:** Demographic features of the participants in baseline

Demographics	HC (***n*** = 80)	MCI (***n*** = 87)	AD dementia (***n*** = 88)	***p*** value
Age(y)	67.3 (4.7)	66.2 (4.3)	67.7 (4.2)	0.785
Sex				
Female	44 (55%)	47 (54%)	50 (47%)	0.931
Male	36 (45%)	40 (46%)	38 (53%)	
Education duration(y)	10.8 (2.9)	10.5 (2.6)	10.4 (2.5)	0.196
ApoE ε4 carrier				
(+)	8 (10%)	15 (17%)	34 (39%)	**0.000**^**b,c**^
(−)	73 (90%)	72 (83%)	54 (61%)	
Mean ChEI dose (mg)	/	/	5.2 (2.3)	/
MMSE	29.3 (0.7)	25.7 (1.4)	17.0 (2.1)	**0.000**^**a,b,c**^
MoCA	26.4 (1.3)	21.6 (1.8)	11.2 (2.5)	**0.000**^**a,b,c**^
SAS	27.4 (3.7)	27.6 (4.5)	27.5 (4.6)	0.986
SDS	29.4 (6.4)	29.1 (6.7)	29.6 (6.5)	0.915
ADAS-cog	7.4 (3.8)	11.8 (4.4)	21.4 (5.2)	**0.000**^**a,b,c**^
AVLT-SR	7.6 (1.3)	5.2 (2.1)	2.3 (1.2)	**0.000**^**a,b,c**^
AVLT-LR	7.3 (1.4)	4.8 (1.6)	1.7 (1.1)	**0.000**^**a,b,c**^
SS-16	11.2 (1.9)	9.1 (2.7)	5.9 (2.7)	**0.000**^**a,b,c**^

“a” means HC group and MCI group are significantly different

“b” means HC group and AD dementia group are significantly different

“c” means MCI group and AD dementia group are significantly different

*Abbreviations*: *MMSE* Mini Mental State Examination, *MoCA* Montreal Cognitive Assessment, *SAS* Zung Self-rating the Anxiety Scale, *SDS* Zung Self-rating Depression Scale, *ADAS-cog* Alzheimer’s Disease Assessment Scale-cognitive subscale, *AVLT* Auditory Verbal Learning test, *SS-16* the 16-item odor identification test from Sniffin Sticks, *HC* healthy control, *MCI* mild cognitive impairment, *AD* Alzheimer’s disease, *ChEI* Cholinesterase inhibitor

### Aβ_1–42_ in plasma NDEs was elevated in MCI and AD dementia patients

**Fig. 1:**
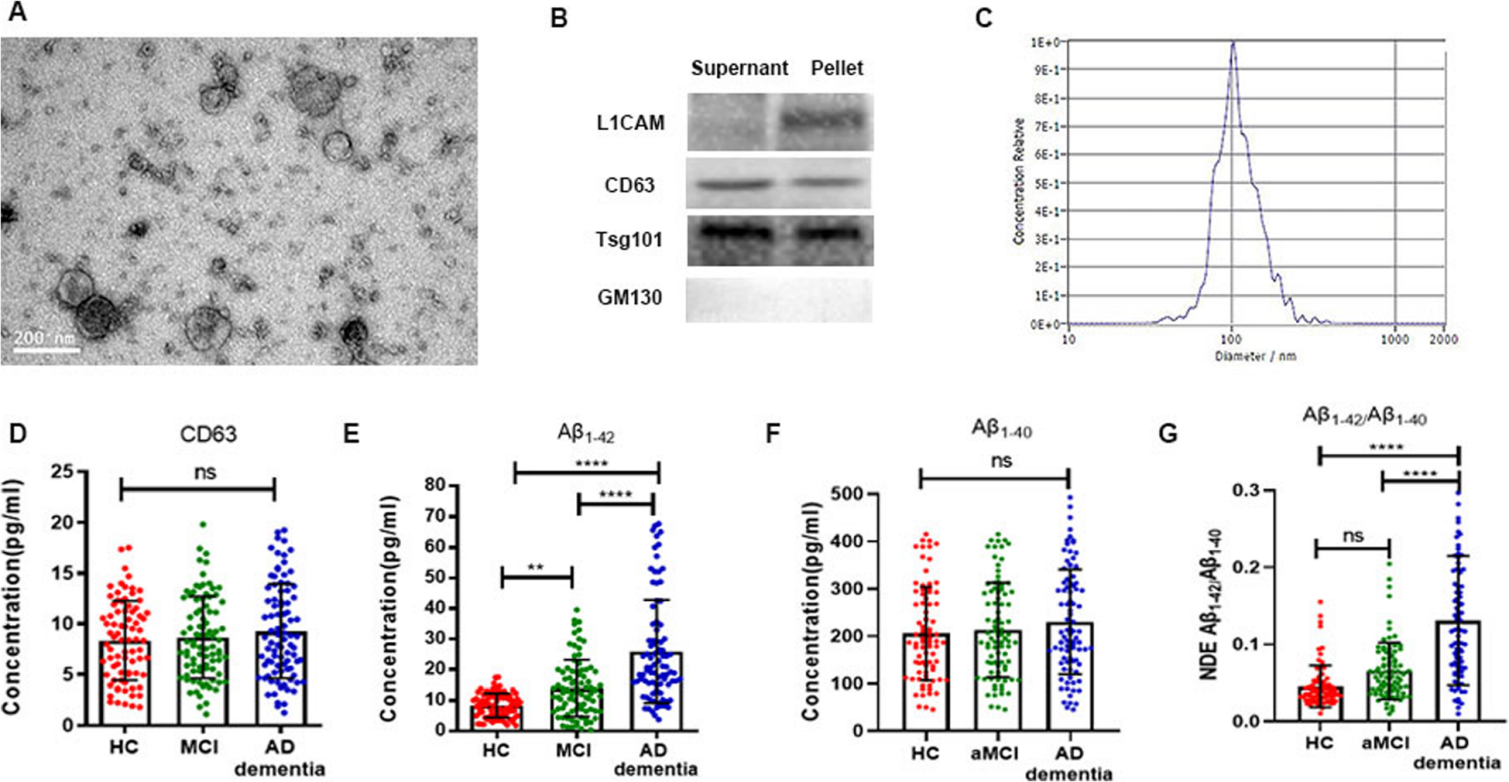
Identification and quantification of neuronal-derived exosomes (NDEs). **a**. Electron microscopic image showing that NDEs were successfully collected. **b**. Western blot showing that exosomal positive marker L1CAM, CD63, Tsg101 was highly expressed in exosomal pellet but not detected in supernatants. **c**.NTA analysis of concentration and diameter in NDEs. **d**.ELISA test of CD63 level in NDEs indicating that the number of exosomes was not significantly different in three groups. **e**. ELISA test of Aβ_1–42_ level in NDEs showing that Aβ_1–42_ was elevated in MCI and AD dementia groups. **: *p* < 0.01; ****: *p* < 0.001. **f**. ELISA test of Aβ_1–40_ level in NDEs indicating that Aβ_1–40_ was not significantly different in three groups. **g**. ELISA test of Aβ_1–42_/Aβ_1–40_ level in NDEs indicating that Aβ_1–42_/Aβ_1–40_ level was elevated in MCI and AD dementia groups. ****: *p* < 0.001

Plasma NDEs were first analyzed for morphology and size distribution using TEM (Fig. 1a), which revealed a population of morphologically distinctive particles of approximately 100-nm diameter, as previously reported [25]. The purity of plasma NDEs was also validated with western blot by three positive exosomal markers (L1CAM, CD63, and Tsg101) and one negative exosomal marker (GM130) (Fig. 1b). In Fig. 1c, the size of the exosomes was directly determined by NTA. Moreover, by performing the ELISA assay, we found the expression of Aβ_1–42_ among three groups based on the consistent distribution of CD63 (Fig. 1d). As shown in Fig. 1e, AD dementia patients exhibited significantly higher concentrations of Aβ_1–42_ in NDE compared to healthy and MCI volunteers (26.0 ± 16.8 pg/ml for AD dementia; 14.0 ± 9.3 pg/ml for MCI, 8.4 ± 3.9 pg/ml for controls, *p* < 0.001). Moreover, MCI patients had greater concentrations of Aβ_1–42_ relative to healthy volunteers (*p* = 0.001). However, there was no significant difference in the Aβ_1–40_ levels among the three groups in Fig. 1f. As for the ratio of Aβ_1–42_/ Aβ_1–40_, AD dementia patients showed elevated Aβ_1–42_/ Aβ_1–40_ ratios than MCI and HC groups (0.13 ± 0.08 for AD dementia; 0.07 ± 0.04 for MCI, 0.05 ± 0.03 for controls, *p* < 0.001) in Fig. 1g. There was only an increasing trend but less statistical significance compared the MCI and HC groups (*p* = 0.057).

### Evaluation of the MCI converters and non-converters

In total, 78 MCI patients completed both the 2 years and 3 years follow-up questionnaires. The mean follow-up time for 2 years and 3 years revisits were 24.3 ± 2.1 and 34.7 ± 3.2 months. 8 of 78 MCI patients (10.3%) developed AD dementia after a median of 2 years of prospective follow-up; whereas 16 of 78 MCI (20.5%) developed AD dementia after a median of 3 years of prospective follow-up. Demographic characteristics of the 3 years’ follow-up visit between MCI converters and non-converters are described in Table 2. All the MCI-c patients met the criteria for probable AD dementia. The MCI-c patients had significantly lower scores on the MMSE, MoCA and AVLT tests than the MCI-nc patients (*p* < 0.001). There were no significant differences in age, sex, or education between the non-converters and converters.

**Table 2:** Characteristics of MCI non-convertors and convertors in three years follow-up

Demographics	MCI-nc (***n*** = 62)	MCI-c (***n*** = 16)	***p*** value
Age, y	68.3 (4.1)	68.5 (3.8)	0.844
Sex			
Female	34 (55%)	9 (56%)	0.919
Male	28 (45%)	7 (44%)	
Education duration, y	10.4 (2.7)	10.3 (1.9)	0.953
ApoE ε4 carrier			
(+)	10 (16%)	4 (25%)	0.410
(−)	52 (84%)	12 (75%)	
MMSE	25.8 (1.4)	19.3 (1.7)	**0.000**
MoCA	22.3 (1.7)	14.5 (1.5)	**0.000**
ADAS-cog	19.2 (3.6)	30.0 (3.3)	**0.000**
AVLT-SR	5.7 (1.4)	2.8 (1.3)	**0.000**
AVLT-LR	4.1 (1.3)	1.5 (1.0)	**0.000**

*Abbreviations*: *MMSE* Mini Mental State Examination, *MoCA* Montreal Cognitive Assessment, *ADAS-cog* Alzheimer’s Disease Assessment Scale-cognitive subscale, *AVLT* Auditory Verbal Learning test, *MCI-nc* not converted from MCI to AD dementia, *MCI-c* converted from MCI to AD dementia

### Risk of SS-16 and Aβ1–42 NDEs in predicting MCI conversion

**Fig. 2:**
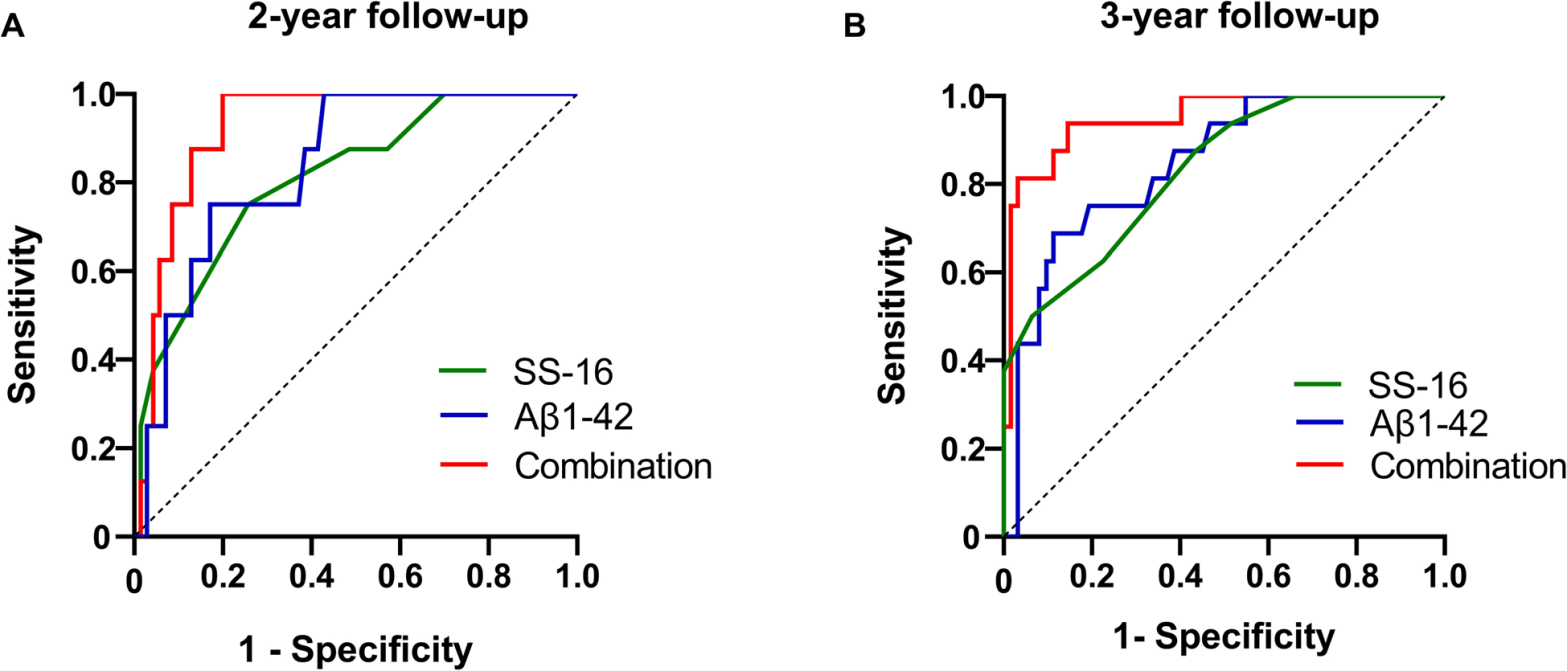
High diagnostic performance of Aβ_1–42_ and SS-16 for MCI converting to AD dementia. **a** showed ROC analysis of Aβ_1–42_ and SS-16 respectively and in combination for MCI-c and MCI-nc in a 2-year follow-up. **b** showed ROC analysis of Aβ_1–42_ and SS-16 respectively and in combination for MCI-c and MCI-nc in a 3-year follow-up

In terms of the predictive power of Aβ_1–42_ and SS-16 for AD dementia, ROC analysis was further conducted to determine whether they have value in predicting the conversion to AD dementia in MCI individuals. As was shown in Fig. 2 and Table 3, SS-16 and plasma Aβ_1–42_ in NDEs classification demonstrated the good value of risk prediction. For the 2-year follow up, the ROC curve showed an AUC of 0.81 with a cutoff value of 8 (*p* = 0.004, 95% CI:0.66–0.96) for SS-16 and 0.84 (*p* = 0.002, 95% CI:0.72–0.95) for Aβ_1–42_ in NDEs with a cutoff value of 14.02, with the combined AUC increased to 0.93 (Table 3). For the 3-year follow-up, AUC was 0.83 (*p* < 0.001, 95% CI:0.72–0.93) for SS-16 and 0.84 (*p* = 0.002, 95% CI:0.72–0.95) for Aβ_1–42_ in NDEs, with the combined AUC of 0.95 (*p* < 0.001, Table 3).

**Table 3:** Characteristics of ROC curves in converted MCI after follow-up visit

	two-years follow up					three-years follow up				
	Sensitivity	Specificity	AUC	95% CI	p	Sensitivity	Specificity	AUC	95% CI	p
SS-16	75.00%	74.29%	0.81	0.66–0.96	0.004	62.50%	77.42%	0.83	0.72–0.93	0.000
Aβ_1–42_	87.50%	61.43%	0.84	0.72–0.95	0.002	87.50%	61.43%	0.84	0.72–0.95	0.000
Combination	87.50%	87.14%	0.93	0.86–0.99	0.000	93.75%	85.48%	0.95	0.89–1.00	0.000

*Abbreviations*: *CI* confidence interval, *SS-16* the 16-item odor identification test from Sniffin Sticks, *ROC* receiver operating characteristic, *AUC* area under the curve

To assess how SS-16 and Aβ_1–42_ increase the risk of AD dementia converting, we built a logistic regression model of the ten markers controlling for age, sex and education. In the 2-year follow-up visit, MCI patients with SS-16 scores lower than 8 showed an 8.3-fold increased risk of converting to AD dementia (*p* = 0.012), whereas patients with higher Aβ_1–42_ levels in NDE showed an 11.1-fold increased risk for developing AD dementia (*p* = 0.028, Table 4). In the 3-year follow up, a similar trend was observed for SS-16 and Aβ_1–42_, where there was a 10-fold risk for individuals with SS-16 scores less than 8(*p* = 0.006) and 8.5-fold risk for Aβ_1–42_ (*p* = 0.002, Table 4). There were no significant differences between ApoE ε4 carriers in the 2-year or 3-year follow-up visit.

**Table 4:** Evaluation between the convertors and non-convertors

	two-years follow up				three-years follow up			
	MCI-nc (***n*** = 70, 90%)	MCI-c (***n*** = 8,10%)	OR (95% CI)	***P***	MCI-nc (***n*** = 62, 79%)	MCI-c (***n*** = 16, 21%)	OR (95% CI)	***P***
SS-16								
< 8	18 (26%)	6 (75%)	0.12 (0.02–0.6)	**0.012**	27 (44%)	14 (88%)	0.1 (0.02–0.5)	**0.006**
≥ 8	52 (74%)	2 (25%)			35 (56%)	2 (12%)		
Aβ_1–42_, pg/ml								
< 14.02	43 (61%)	7 (88%)	11.1 (1.3–95.7)	**0.028**	41 (66%)	3 (19%)	8.5 (2.2–33)	**0.002**
≥ 14.02	27 (39%)	1 (12%)			21 (34%)	13 (81%)		
ApoE ε4 carrier								
(+)	12 (17%)	2 (25%)	1.6 (0.29–9.0)	0.590	10 (16%)	4 (25%)	1.7 (0.5–6.5)	0.415
(−)	58 (83%)	6 (75%)			52 (84%)	12 (75%)		

All results were adjusted for age and sex

*Abbreviations*: *SS-16* the 16-item odor identification test from Sniffin Sticks

### Stratified analysis of SS-16 and Aβ1–42 NDEs in ApoE ε4 status

According to the baseline characteristics, 14 (17.9%) patients were ApoE ε4 positive, we, therefore, investigated the potential predictors of disease progression in specific subgroups. We found that ApoE ε4 non-carriers with lower SS-16 scores (OR = 7.1, 95% CI: 1.4–33.3, *p* = 0.015; Table 5) were more likely to develop AD dementia. Moreover, patients without the ApoE ε4 mutation exhibited higher Aβ_1–42_ levels and showed a higher risk of developing AD dementia (OR = 9.4, 95% CI: 1.9–47.8, *p* = 0.007; Table 5). However, this was not statistically significant and a larger sample size may be needed.

**Table 5:** Analysis of potential predictors in populations with or without ApoE ε4carrier in three-years follow up

	ApoE ε4 carriers (*n* = 14)				ApoE ε4 non-carriers (*n* = 64)			
	MCI-nc (*n* = 10)	MCI-nc (*n* = 4)	OR (95%CI)	*P*	MCI-nc (*n* = 52)	MCI-nc (*n* = 12)	OR (95%CI)	*P*
SS-16								
< 8	6	4	/	/	21	10	0.14 (0.03–0.7)	**0.015**
≥ 8	4	0			31	2		
Aβ_1–42_, pg/ml								
< 14.02	7	1	7 (0.5–97.8)	0.15	34	2	9.4 (1.9–47.8)	**0.007**
≥ 14.02	3	3			18	10		

All results were adjusted for age and sex

*Abbreviations*: *SS-16* the 16-item odor identification test from Sniffin Sticks

### Association between Aβ_1–42_, SS-16 and cognitive function

**Fig. 3:**
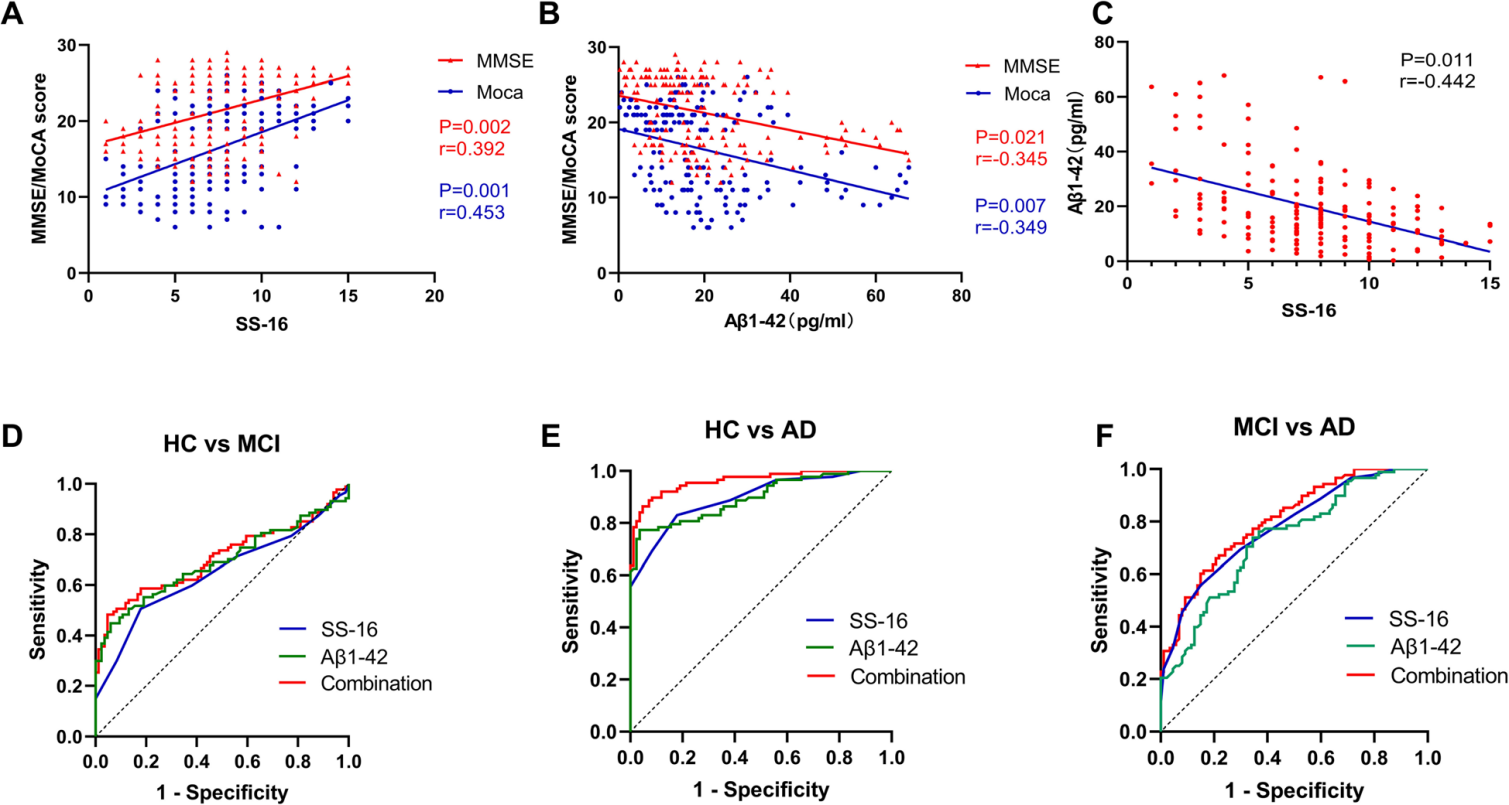
Association Aβ_1–42_, SS-16, and cognitive function. Showed an association between SS-16 and MMSE/MoCA scores in MCI and AD dementia groups. **a**. showed an association between Aβ_1–42_ and MMSE/MoCA scores in MCI and AD dementia groups. **b**. showed an association between SS-16 and Aβ_1–42_ level in MCI and AD dementia groups. ***d-f****. showed the high diagnostic performance of* Aβ_1–42_ and SS-16 respectively and in combination for HC and MCI(**d**), HC and AD dementia (**e**), MCI and AD dementia (**f**)

To determine the association between Aβ_1–42_, SS-16 and MMSE/MoCA, correlation analysis was used. The unadjusted analyses showed a strong association between higher MMSE and MoCA score and higher SS-16 score (*p* = 0.002, r = 0.392 for MMSE; *p* = 0.001, r = 0.453 for MoCA; Fig. 3a) in MCI and AD dementia groups. Similarly, significant negative correlations were found between MMSE/MoCA scores and Aβ_1–42_ levels (*p* = 0.021, r = − 0.345 for MMSE; *p* = 0.007, r = − 0.349 for MoCA; Fig. 3b). Interestingly, further analyses also showed an association between higher SS-16 score and reduced values of Aβ1–42 in NDEs (*p* = 0.011, r = − 0.442; Fig. 3c).

Subsequently, ROC curve analysis was performed to evaluate the discriminative power of Aβ_1–42_ and SS-16 in the diagnosis for MCI and AD dementia, respectively and in combination. Both Aβ_1–42_ (AUC: 0.69; *p* < 0.001; 95% CI: 0.61–0.77 for MCI; AUC: 0.90; *p* < 0.001; 95% CI: 0.85–0.94 for AD dementia, Fig. 3d and e) and SS-16 have diagnostic value for individuals with MCI and patients with AD dementia (AUC: 0.65: *p* = 0.001; 95% CI: 0.56–0.73 for MCI; AUC: 0.90; *p* < 0.001; 95% CI: 0.85–0.94 for AD dementia, Fig. 3d and e). Moreover, their combination resulted in a significant increase in the c-statistics of AUC: 0.71 (95% CI: 0.63–0.79, *p* < 0.001) and AUC: 0.96 (95% CI: 0.94–0.99, *p* < 0.001), which showed better diagnostic efficiency for MCI or AD dementia relative to the use of each of these variables in isolation (Fig. 3d and e). Moreover, the combination of Aβ_1–42_ and SS-16 also showed modest accuracy in distinguishing MCI and AD dementia groups (AUC: 0.81; *p* < 0.001; 95% CI: 0.74–0.87, Fig. 3f).

## Discussion

Previous studies have identified biomarkers to facilitate identifying individuals who are at risk of developing AD dementia. Our study was a longitudinal study that investigated whether NDEs in plasma and olfactory tests may also predict the conversion from MCI to AD dementia in a Chinese population. During the 3-year follow-up, 16 subjects developed probable AD dementia (MCI-c) and 62 did not convert to AD dementia (MCI-nc). At baseline, there were significant differences in SS-16 scores and *neurogenic exosomal* Aβ_1–42_ levels between individuals who later developed dementia relative to those who did not. Our study indicated that age, ApoE ε4 status, higher-levels of Aβ_1–42_ in plasma NDEs and lower SS-16 scores predicted the AD dementia conversion in individuals with MCI patients with modest accuracy.

Olfactory impairment was first reported as a clinical symptom of AD dementia more than 30 years ago [26]. Our finding that olfactory function was impaired and predicted transition is consistent with previous literature showing that olfactory deficiencies exist before patients are diagnosed with AD [27] and literature showing that it predicts the conversion of MCI to AD dementia [28]. In autopsy studies [29], the absence of odor identification was associated with plaques and tangles in the olfactory bulb, entorhinal cortex and cornu ammonis 1 regions of the hippocampus. The SS-16 test was validated as a diagnostic tool for AD dementia and MCI patients in our study. In this study, level of Aβ_1–42_ in NDEs increased the risk of developing AD dementia in individuals with MCI in the 2 and 3-year follow-up. Compared to the 2-year follow-up, the level of Aβ_1–42_ showed better predictive power for the cognitive decline in MCI individuals in a 3-year revisit. It was believed that the combination of P-tau and Aβ_1–42_ in CSF had the greatest predictive accuracy for predicting the conversion from MCI to dementia [30, 31]. A recent study also showed that plasma neuronal-derived exosomal Aβ_1–42_, T-tau, and P-T181-tau had the same capacity as those in CSF for the diagnosis of AD dementia and MCI [32].

In our study, plasma NDEs Aβ_1–42_ differentiated between cognitive controls, MCI patients and AD dementia patients; and predicted the risk of MCI progressing to AD dementia in the longitudinal study. Neuronal exosomes containing Aβ-peptide products transmit Aβ to adjacent cells, other brain regions and circulatory systems, indicating that neuronal exosomes extracted from plasma or CSF can specifically evaluate the relevant neuropathological processes in the CNS [33]. Moreover, NDEs may act as vehicles for the neuron-to-neuron transfer of Aβ oligomers in a prion-like manner [34]. The propagation of Aβ in the brain from NDEs could also serve as a potential treatment target by inhibiting either formation, secretion, or cellular uptake of exosomes [35]. Our findings suggested that a combination of the SS-16 with that plasma NDEs Aβ_1–42_ helped screen cognitively healthy individuals and MCI patients. Our findings identify that these biomarkers may be beneficial in identifying at risk individuals which may be helpful for the development of preventative medicine. The mechanisms underlying the association between exosomes and olfactory function are complex. Many studies have shown that the relationship between Aβ alters the connectivity of the peripheral olfactory neural circuit even before the onset of amyloid plaques [36]. The oligomeric amyloid-β peptide affects the responses of mitral cells (MCs) in the rat olfactory bulb [37]. Impaired blood–brain barrier (BBB) may lead to disruptions in CSF flow through the olfactory system, resulting in the less efficient removal of Aβ from the CNS [38]. What’s more, exosomes played an important role in amyloid Aβ clearance in CNS [39]. Hence, further research is needed to understand the relationship between exosomes and olfactory function.

Some limitations of our study should be considered when interpreting the results. First, we chose to use only one odor identification test to make the study more clinically feasible. Previous studies have indicated that patients with AD dementia have a higher olfactory threshold compared to healthy controls [40]. For individuals with extremely high olfactory thresholds, the odor discrimination test results may also be affected. Therefore, if conditions permit, it would be preferable to administer all three parts of the standard SS-16 to obtain the maximum amount of reliable data to fully evaluate olfactory function. Secondly, Aβ_1–40_ in NDEs revealed no significant difference between the MCI and healthy controls, possibly due to the small number of cases. To further the understanding of how exosomes predict MCI transformation, future studies using larger sample sizes are needed. What’s more, it is needed to verify whether the predictive power of SS-16 and NDE Aβ1–42 is specific to AD dementia, considering that MCI is also associated with other kinds of dementia, such as dementia with Lewy bodies (DLB). Hence, it is necessary to include a relatively large sample size and used a longitudinal design and long time-points to verify the prediction of olfaction and Aβ1–42 in NDEs with respect to conversion toward AD dementia or DLB in MCI patients.

In conclusion, our findings suggest that impaired olfaction and plasma NDE cargo proteins traffic from the CNS to blood are shown in MCI and they predict the conversion from MCI to AD dementia. Our findings highlight the clinical utility of these biomarkers to identifying at risk individuals. Further work is needed to identify whether modulating these early abnormalities may prevent the progression of MCI to AD dementia.

## Supplementary information

**Additional file 1: Table S1.** Single-question-score of SS-16 among groups in baseline. **Table S2.** Characteristics of ROC curves of SS-16 and Aβ1–42 in NDEs among groups. **Table S3.** Single-question-score of SS-16 between convertors and non-convertors in three-years follow up.

## Data Availability

All data generated or analysed during this study are included in this published article.
