# Supplementary material for: Increased prediction value of biomarker combinations for the conversion of mild cognitive impairment to Alzheimer’s dementia
Source: Transl Neurodegener. 2020 Aug 3;9:30. doi: 10.1186/s40035-020-00210-5 (PMC7397685; doi:10.1186/s40035-020-00210-5)
Supplement: Supplementary file 1 — Additional file 1: Table S1. Single-question-score of SS-16 among groups in baseline. Table S2. Characteristics of ROC curves of SS-16 and Aβ1–42 in NDEs among groups. Table S3. Single-question-score of SS-16 between convertors and non-convertors in three-years follow up. [file 40035_2020_210_MOESM1_ESM.doc]

**Table S1. Single-question-score of SS-16 among groups in baseline**

| **Items** | **SS-16** | **HC (n=80)** | **MCI (n=87)** | **AD (n=88)** | **p value** |
| --- | --- | --- | --- | --- | --- |
| 1 | Orange | 80.0% | 60.0% | 36.4% | **0.000^b,c^** |
| 2 | Leather | 95.2% | 78.2% | 40.0% | **0.000^b,c^** |
| 3 | Cinnamon | 73.8% | 52.9% | 35.2% | **0.000 ^b,c^** |
| 4 | Peppermint | 95.2% | 63.2% | 31.8% | **0.000^a,b,c^** |
| 5 | Banana | 78.5% | 60.9% | 41.4% | **0.002 ^b,c^** |
| 6 | Lemon | 84.5% | 66.7% | 55.7% | **0.000^b,c^** |
| 7 | Liquorice | 70.2% | 62.1% | 45.5% | **0.008^b,c^** |
| 8 | Turpentine | 70.2% | 64.3% | 48.9% | **0.019 ^b,c^** |
| 9 | Garlic | 78.5% | 79.3% | 53.4% | **0.000^b,c^** |
| 10 | Coffee | 88.1% | 73.6% | 46.6% | **0.000^b,c^** |
| 11 | Apple | 22.6% | 26.4% | 20.5% | 0.550 |
| 12 | Cloves | 65.5% | 67.8% | 38.6% | **0.035 ^b,c^** |
| 13 | Pneapple | 71.4% | 56.3% | 36.0% | **0.000^a,b,c^** |
| 14 | Rose | 68.0% | 69.0% | 53.4% | 0.064 |
| 15 | Anise | 67.9% | 67.8% | 47.7% | **0.014^b,c^** |
| 16 | Fish | 95.2% | 83.9% | 56.8% | **0.000^b,c^** |

**Abbreviations:** HC: healthy control, MCI: mild cognitive impairment, AD: Alzheimer’s disease, SS-16: the 16-item odor identification test from Sniffin Sticks; “a” means HC group and MCI group are significantly different using split Chi-square; “b” means HC group and AD group are significantly different using split Chi-square;“c” means MCI group and AD group are significantly different using split Chi-square.

|  |  | Sensitivity | Specificity | AUC | 95% CI | | p | Cut-off level |
| --- | --- | --- | --- | --- | --- | --- | --- | --- |
| HC vs MCI | SS-16 | 59.77% | 61.90% | 0.65 | 0.565 | 0.73 | 0.001 | 9 |
|  | Aβ_1-42_ | 67.82% | 54.76% | 0.69 | 0.61 | 0.77 | 0.000 | 8.73pg/ml |
|  | Combination | 67.82% | 57.14% | 0.71 | 0.63 | 0.79 | 0.000 | - |
| HC vs AD | SS-16 | 82.95% | 82.14% | 0.90 | 0.85 | 0.94 | 0.000 | 8 |
|  | Aβ_1-42_ | 80.68% | 80.95% | 0.90 | 0.85 | 0.94 | 0.000 | 12.05 pg/ml |
|  | Combination | 84.09% | 95.24% | 0.96 | 0.94 | 0.99 | 0.000 | - |
| MCI vs AD | SS-16 | 69.32% | 70.11% | 0.78 | 071 | 0.85 | 0.000 | 7 |
|  | Aβ_1-42_ | 73.86% | 65.52% | 0.73 | 0.66 | 0.81 | 0.000 | 15.54 pg/ml |
|  | Combination | 77.27% | 65.52% | 0.81 | 0.74 | 0.87 | 0.000 | - |

**Table S2. Characteristics of ROC curves of SS-16 and Aβ1-42 in NDEs among groups**

**Abbreviations:** ROC: Receiver operating characteristic; HC: healthy control, MCI: mild cognitive impairment, AD: Alzheimer’s disease, SS-16: the 16-item odor identification test from Sniffin Sticks.

|  |  | **MCI-nc (n = 62)** | |  | **MCI-c (n = 16)** | | **OR (95% CI)** | ***P*** |
| --- | --- | --- | --- | --- | --- | --- | --- | --- |
| **Items** | **SS-16** | **YES** | **NO** |  | **YES** | **NO** |
| 1 | Orange | 38 | 24 |  | 9 | 7 | 0.8(0.3-2.5) | 0.71 |
| 2 | Leather | 35 | 27 |  | 8 | 8 | 0.8(0.3-2.3) | 0.64 |
| 3 | Cinnamon | 33 | 29 |  | 5 | 11 | 0.4(0.1-1.3) | 0.12 |
| 4 | **Peppermint** | 49 | 13 |  | 4 | 12 | 0.09(0.02-0.3) | **<0.001** |
| 5 | Banana | 39 | 23 |  | 10 | 6 | 1.0(0.3-3.2) | 0.99 |
| 6 | Lemon | 33 | 29 |  | 7 | 9 | 0.7(0.2-2.1) | 0.50 |
| 7 | Liquorice | 37 | 25 |  | 6 | 10 | 0.4(0.1-1.3) | 0.12 |
| 8 | Turpentine | 34 | 28 |  | 6 | 10 | 0.5(0.2-1.5) | 0.22 |
| 9 | **Garlic** | 41 | 21 |  | 6 | 10 | 0.3(0.1-1.0) | **0.043** |
| 10 | Coffee | 35 | 27 |  | 9 | 7 | 1.0(0.3-3.0) | 0.99 |
| 11 | Apple | 12 | 50 |  | 3 | 13 | 1.0(0.2-4.2) | 0.97 |
| 12 | Cloves | 42 | 20 |  | 8 | 8 | 0.5(0.2-1.5) | 0.19 |
| 13 | **Pineapple** | 42 | 20 |  | 3 | 13 | 0.1(0.03-0.4) | **0.002** |
| 14 | Rose | 32 | 30 |  | 9 | 7 | 1.2(0.4-3.6) | 0.74 |
| 15 | Anise | 37 | 25 |  | 6 | 10 | 0.4(0.1-1.3) | 0.12 |
| 16 | **Fish** | 52 | 10 |  | 6 | 10 | 0.1(0.03-0.3) | **<0.001** |

**Table S3. Single-question-score of SS-16 between convertors and non-convertors in three-years follow up**

**Abbreviations:** SS-16: the 16-item odor identification test from Sniffin Sticks. All results represented were adjusted for age and sex.
